# Machine Learning-Assisted Ensemble Analysis for the Prediction of Response to Neoadjuvant Chemotherapy in Locally Advanced Cervical Cancer

**DOI:** 10.3389/fonc.2022.817250

**Published:** 2022-03-29

**Authors:** Yibao Huang, Qingqing Zhu, Liru Xue, Xiaoran Zhu, Yingying Chen, Mingfu Wu

**Affiliations:** Department of Gynecology, National Clinical Research Center for Obstetrical and Gynecological Diseases, Key Laboratory of Cancer Invasion and Metastasis, Ministry of Education, Tongji Hospital, Tongji Medical College, Huazhong University of Science and Technology, Wuhan, China

**Keywords:** locally advanced cervical cancer, neoadjuvant chemotherapy, machine learning analysis, predictive model, pathology response

## Abstract

The clinical benefit of neoadjuvant chemotherapy (NACT) before concurrent chemoradiotherapy (CCRT) vs. adjuvant chemotherapy after CCRT is debated. Non-response to platinum-based NACT is a major contributor to poor prognosis, but there is currently no reliable method for predicting the response to NACT (rNACT) in patients with locally advanced cervical cancer (LACC). In this study we developed a machine learning (ML)-assisted model to accurately predict rNACT. We retrospectively analyzed data on 636 patients diagnosed with stage IB2 to IIA2 cervical cancer at our hospital between January 1, 2010 and December 1, 2020. Five ML-assisted models were developed from candidate clinical features using 2-step estimation methods. Receiver operating characteristic curve (ROC), clinical impact curve, and decision curve analyses were performed to evaluate the robustness and clinical applicability of each model. A total of 30 candidate variables were ultimately included in the rNACT prediction model. The areas under the ROC curve of models constructed using the random forest classifier (RFC), support vector machine, eXtreme gradient boosting, artificial neural network, and decision tree ranged from 0.682 to 0.847. The RFC model had the highest predictive accuracy, which was achieved by incorporating inflammatory factors such as platelet-to-lymphocyte ratio, neutrophil-to-lymphocyte ratio, neutrophil-to-albumin ratio, and lymphocyte-to-monocyte ratio. These results demonstrate that the ML-based prediction model developed using the RFC can be used to identify LACC patients who are likely to respond to rNACT, which can guide treatment selection and improve clinical outcomes.

## Introduction

Cervical cancer is a malignant tumor and major cause of morbidity and mortality, with an estimated 500,000 new cases and 300,000 deaths each year (1, 2). Over the past decade, substantial progress has been made in the early diagnosis and treatment of locally advanced cervical cancer (LACC) (3). The standard treatments for LACC are surgery, radiation therapy, and chemotherapy (CT) (3), but none of these are optimal. Local residual disease following chemoradiotherapy (CRT) can be treated by salvage surgery; however, this is associated with various complications (4, 5). Additionally, the response of LACC patients to radical surgery after radiotherapy and CT is generally poor, while radiotherapy may not be a treatment option in low-income countries (6, 7). As such, there is a need for more effective and accessible treatment options for LACC.

As a potential alternative therapy, platinum-based neoadjuvant (NA)CT has been shown to reduce tumor volume (3, 8). According to the International Federation of Gynecology and Obstetrics (FIGO) classification system of 2009, NACT can be considered for patients with stage IB2 to IIA2 LACC, especially before radical hysterectomy (9–11). Cisplatin-based NACT is associated with improved long-term survival rates in LACC (12–14). However, there are currently no models that can accurately predict the pathologic response to NACT in these patients, although this could facilitate clinical management. Machine learning (ML) is a data analysis method with applications in healthcare (15). Compared to conventional statistical models, ML-based ensemble analysis can ensure robustness of a statistical model and improve its predictive accuracy through iterative algorithms.

There is no consensus on the cutoff for optimal response to NACT (rNACT) in patients with LACC, with overall response rates to NACT ranging from 52 to 95% (16, 17). In this study, we applied ML-based algorithms to establish a model to accurately predict rNACT in LACC patients by using preoperative clinical parameters and inflammatory markers.

## Methods

### Patient Selection

**Figure 1:**
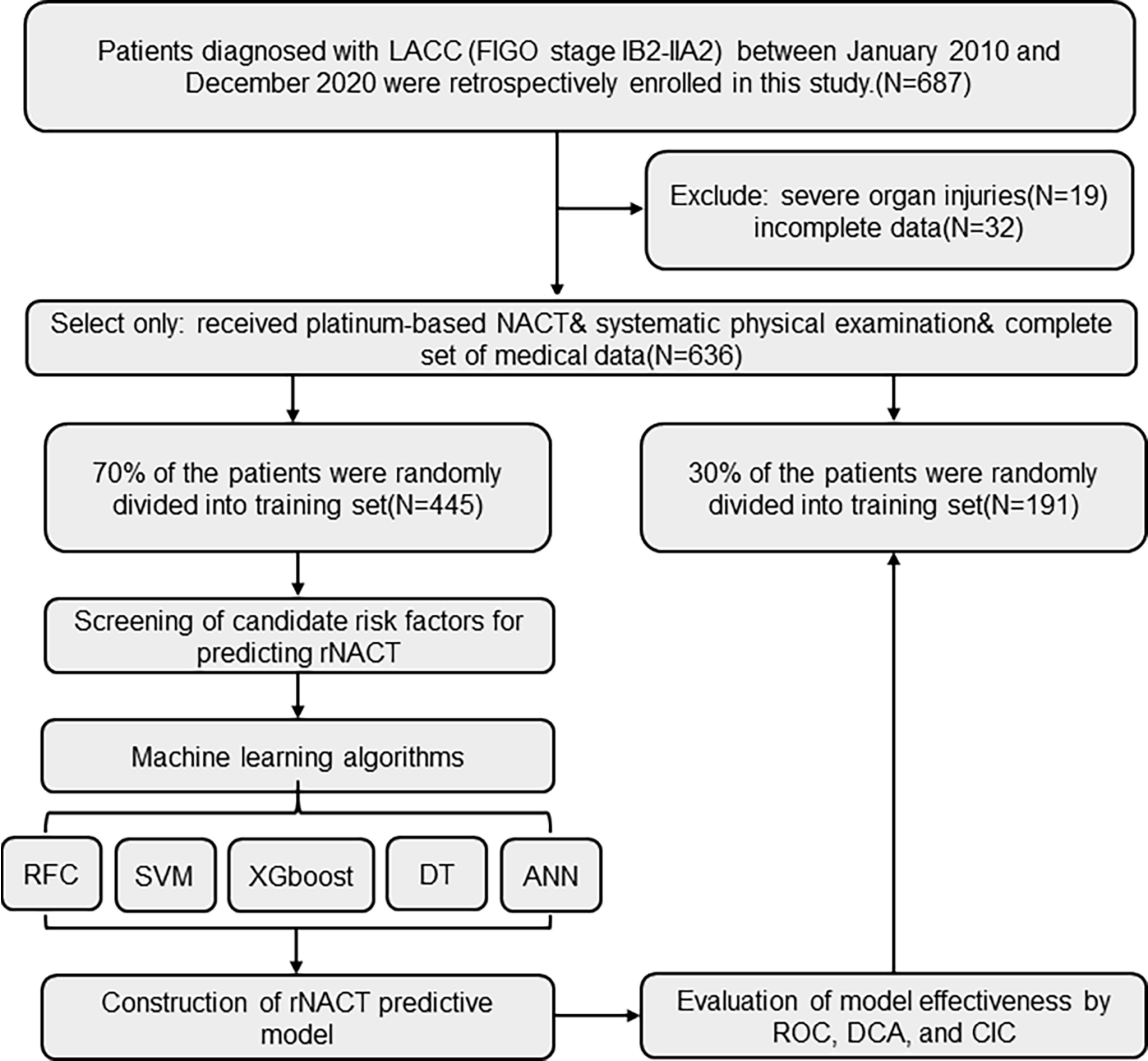
Flow diagram of patient selection and data processing. ANN, artificial neural network; CIC, clinical impact curve; DCA, decision curve analysis; DT, decision tree; FIGO, International Federation of Gynecology and Obstetrics; RFC, random forest classifier; ROC, receiver operating characteristic; SVM, support vector machine; XGboost, eXtreme gradient boosting.

Patients who were diagnosed with FIGO stage IB2–IIA2 cervical cancer at the Tongji Hospital of Tongji Medical College, Huazhong University of Science and Technology (Wuhan, China) between January 2010 and December 2020 were retrospectively enrolled in this study. The inclusion criteria for patients were as follows: (i) received platinum-based NACT, without adverse effects; (ii) received a standard cycle of NACT before the operation with no other treatment; (iii) underwent systematic physical examination before the operation, including peripheral blood monitoring and imaging examination; and (iv) a complete set of medical data was available. We excluded patients who had severe organ injuries or incomplete clinical parameters, laboratory test results, and imaging findings in their medical records. The study protocol was in compliance with the provisions of the Helsinki Declaration (2013 revision) and was approved by the Institutional Review Committee of Tongji Hospital, Tongji Medical College, Huazhong University of Science and Technology (TJ-IRB20210631). All information of the patients was strictly confidential and informed consent was waived due to its traceability. The workflow for LACC patient selection and model construction is summarized in **Figure 1**. The study was approved by the Institutional Review Committee of Tongji Hospital, Tongji Medical College, Huazhong University of Science and Technology (TJ-IRB20210631).

### Data Collection and Quality Assessment

The following data were collected for all patients: age, body mass index, histology, FIGO stage, tumor size before NACT, histologic grade, lymph node metastasis, lymph vascular space invasion, parametrial involvement, surgical margin, neutrophil count (10^9^/l), lymphocyte count (10^9^/l), platelet count (10^9^/l), monocyte count (10^9^/l), hemoglobin, albumin, globulin, and tumor markers. For variables with missing values, the median was typically used. If ≥10% of values were missing for a given variable, it was excluded from variable screening for the final model.

### Evaluation Criteria for NACT

For pathologic response assessment, all patients who received NACT were independently examined by 2 pathologists. rNACT was evaluated according to the Response Evaluation Criteria in Solid Tumors (RECIST) v1.1 criteria (18), and was categorized into the following 4 levels according to the presence or absence of pathologic response: (i) complete response (CR), almost complete disappearance of cancer lesions; (ii) partial response (PR), ≥30% decrease in total maximum diameter of cancer lesions; (iii) progressive disease (PD), ≥20% decrease in total maximum diameter of cancer lesions; and (iv) stable disease (SD), total maximum diameter of cancer lesions defined as either insufficient contraction in line with PR or increased compliance with PD. LACC patients were considered to be rNACT if they were determined as having CR or PR following NACT treatment; meanwhile, patients with SD or PD were regarded as non-rNACT.

### Development and Validation of ML-Based Models

Four ML-based algorithms were used to build predictive models. We used the Classification and Regression Training (caret) package to randomly divide the dataset into 2 parts, 70% for model training and 30% for model validation. A total of 5 ML-based algorithms were evaluated for the predictive model. The model variables were screened by 2-step estimation (19) according to the following formula.


$$\hat{m}={\hat{m}}_{3}\cap {\hat{m}}_{4}$$

The characteristic variable was X and the target variable was Y; these were evenly divided into 2 parts—namely, X1, Y1 and X2, Y2. Through univariate screening, the variable quantum set m^1^ was screened on X1 and Y1, and m^2^ was filtered by X2 and Y2. A lasso was then used to re-fit the model, and the filtered variables were marked as m^3^ and m^4^. The optimal subset for modeling was obtained based on the intersection of the variable sets. The model was evaluated by inspection, discrimination, and calibration. A receiver operating characteristic (ROC) curve was used to assess the predictive accuracy of the model in the training and validation sets. The discriminatory ability of each model was quantified by the area under the ROC curve (AUC), decision curve analysis (DCA), and clinical impact curve (CIC) analysis.

### Statistical Analysis

Median (interquartile range) and frequencies (%) were described for continuous and categorical variables, respectively. The chi-squared test or Mann–Whitney U test was used to compare baseline clinical characteristics between the rNACT and non-rNACT cohorts as appropriate. All analyses were performed using Python v3.9.2 (https://www.python.org/) and R v4.0.4 (http://www.r-project.org/). All tests were 2-sided, and p <0.05 was considered statistically significant.

## Results

### Baseline Characteristics of the Study Population and rNACT

The detailed clinical characteristics and pathologic baseline data of 636 patients with LACC are shown in **Table 1**. All patients received platinum-based NACT, with no serious adverse reactions. For internal validation, patients were randomly divided into a training set (N = 445, 70%) and validation set (N = 191, 30%) using the caret package. According to the RECIST criteria, 396 (88.9%) and 162 (84.8%) patients showed rNACT in the training and validation sets, respectively, indicating that these patients were sensitive to NACT. Follow-up treatment was determined according to the condition of the patients and included radical surgery, radiation, and concurrent (C)CRT. Of these patients, 614 (96.6%) underwent radical surgery and 91 (14.3%) received radiotherapy or CCRT.

**Table 1:** Baseline demographic and clinicopathologic features of patients with LACC with and without a diagnosis of rNACT.

Variables	Overall	Training set		P-value	Testing set		P-value
	N = 636	Responders (N = 396)	Non-responders (N = 49)		Responders (N = 162)	Non-responders (N = 29)	
Age, years	47.00 [42.00, 52.00]	47.00 [42.75, 52.00]	49.00 [41.00, 54.00]	0.713	47.00 [43.00, 52.00]	45.00 [37.00, 52.00]	0.229
Weight, kg	55.44 [50.00, 60.00]	55.44 [50.00, 60.00]	55.44 [49.00, 59.00]	0.563	55.44 [50.00, 60.00]	55.00 [53.00, 60.00]	0.642
Height, m	157.40 [154.00, 160.00]	157.40 [154.00, 160.62]	157.40 [155.00, 160.00]	0.86	157.40 [155.00, 160.00]	158.00 [154.00, 160.00]	0.934
Smoking							
Yes	34 (5.3)	22 (5.6)	1 (2.0)	0.48	10 (6.2)	1 (3.4)	0.883
No	602 (94.7)	374 (94.4)	48 (98.0)		152 (93.8)	28 (96.6)	
FIGO stage							
Ib	4 (0.6)	2 (0.5)	1 (2.0)	0.356	1 (0.6)	0 (0.0)	0.845
Ib2	402 (63.2)	256 (64.6)	28 (57.1)		99 (61.1)	19 (65.5)	
IIa	12 (1.9)	7 (1.8)	2 (4.1)		3 (1.9)	0 (0.0)	
IIa2	218 (34.3)	131 (33.1)	18 (36.7)		59 (36.4)	10 (34.5)	
Histology							
SCC	542 (85.2)	340 (85.9)	44 (89.8)	0.235	134 (82.7)	24 (82.8)	0.921
ADC	68 (10.7)	42 (10.6)	3 (6.1)		19 (11.7)	4 (13.8)	
AdCa	9 (1.4)	5 (1.3)	2 (4.1)		2 (1.2)	0 (0.0)	
Other	17 (2.7)	9 (2.3)	0 (0.0)		7 (4.3)	1 (3.4)	
Tumor grade							
G1	37 (5.8)	28 (7.1)	1 (2.0)	0.063	7 (4.3)	1 (3.4)	0.04
G2	278 (43.7)	186 (47.0)	19 (38.8)		64 (39.5)	9 (31.0)	
G3	241 (37.9)	138 (34.8)	26 (53.1)		59 (36.4)	18 (62.1)	
Unknown	80 (12.6)	44 (11.1)	3 (6.1)		32 (19.8)	1 (3.4)	
Tumor size	3.60 [2.50, 4.10]	3.50 [2.30, 4.00]	6.00 [5.00, 7.00]	<0.001	3.50 [2.50, 4.00]	6.00 [5.00, 7.00]	<0.001
Lymphatic invasion							
Yes	85 (13.4)	56 (14.1)	12 (24.5)	0.152	13 (8.0)	4 (13.8)	0.597
No	528 (83.0)	326 (82.3)	36 (73.5)		142 (87.7)	24 (82.8)	
Unknown	23 (3.6)	14 (3.5)	1 (2.0)		7 (4.3)	1 (3.4)	
Parametrial invasion							
Yes	38 (6.0)	21 (5.3)	7 (14.3)	0.033	7 (4.3)	3 (10.3)	0.374
No	598 (94.0)	375 (94.7)	42 (85.7)		155 (95.7)	26 (89.7)	
Pelvic lymph metastasis							
Yes	103 (16.2)	56 (14.1)	22 (44.9)	<0.001	18 (11.1)	7 (24.1)	0.159
No	510 (80.2)	326 (82.3)	26 (53.1)		137 (84.6)	21 (72.4)	
Unknown	23 (3.6)	14 (3.5)	1 (2.0)		7 (4.3)	1 (3.4)	
Paraortic lymph metastasis							
Yes	18 (2.8)	11 (2.8)	4 (8.2)	0.128	3 (1.9)	0 (0.0)	0.74
No	595 (93.6)	371 (93.7)	44 (89.8)		152 (93.8)	28 (96.6)	
Unknown	23 (3.6)	14 (3.5)	1 (2.0)		7 (4.3)	1 (3.4)	
SCC	4.30 [0.80, 4.30]	4.30 [0.80, 4.30]	2.20 [0.60, 4.30]	0.041	4.30 [1.12, 4.30]	1.30 [0.70, 4.30]	0.028
P53							
Positive	32 (5.0)	16 (4.0)	1 (2.0)	0.382	11 (6.8)	4 (13.8)	0.282
Negative	14 (2.2)	11 (2.8)	0 (0.0)		2 (1.2)	1 (3.4)	
Unknown	590 (92.8)	369 (93.2)	48 (98.0)		149 (92.0)	24 (82.8)	
CEA	5.15 [3.44, 5.15]	5.15 [3.10, 5.15]	5.15 [3.61, 5.15]	0.705	5.15 [4.16, 5.15]	5.15 [5.15, 5.15]	0.96
CA125	42.00 [42.00, 42.00]	42.00 [42.00, 42.00]	42.00 [42.00, 42.00]	0.008	42.00 [28.73, 42.00]	42.00 [42.00, 42.00]	0.289
CA199	32.19 [32.19, 32.19]	32.19 [32.19, 32.19]	32.19 [32.19, 32.19]	0.605	32.19 [28.69, 32.19]	32.19 [32.19, 32.19]	0.521
CCRT							
Yes	91 (14.3)	16 (4.0)	44 (89.8)	<0.001	9 (5.6)	22 (75.9)	<0.001
No	530 (83.3)	371 (93.7)	5 (10.2)		147 (90.7)	7 (24.1)	
Unknown	15 (2.4)	9 (2.3)	0 (0.0)		6 (3.7)	0 (0.0)	
Surgical method							
Laparoscopy	485 (76.3)	300 (75.8)	38 (77.6)	0.888	127 (78.4)	20 (69.0)	0.424
Open	129 (20.3)	83 (21.0)	10 (20.4)		28 (17.3)	8 (27.6)	
Unknown	22 (3.5)	13 (3.3)	1 (2.0)		7 (4.3)	1 (3.4)	
Surgical margin							
Positive	11 (1.7)	4 (1.0)	2 (4.1)		4 (2.5)	1 (3.4)	
Negative	603 (94.8)	379 (95.7)	46 (93.9)	0.194	151 (93.2)	27 (93.1)	0.935
Positive	22 (3.5)	13 (3.3)	1 (2.0)		7 (4.3)	1 (3.4)	
LND	31.00 [25.00, 38.00]	31.00 [25.00, 38.00]	32.00 [27.00, 37.00]	0.309	32.00 [25.00, 39.75]	30.00 [26.00, 34.00]	0.302
Platelet count	251.50 [209.00, 276.40]	239.00 [204.75, 276.40]	327.00 [301.00, 370.00]	<0.001	245.50 [204.50, 276.00]	347.00 [318.00, 386.00]	<0.001
Leukocyte count	6.30 [4.92, 7.43]	6.36 [5.03, 7.43]	5.15 [4.17, 7.43]	0.01	6.37 [4.89, 7.43]	6.02 [5.14, 6.53]	0.351
Lymphocyte count	1.63 [1.27, 1.77]	1.63 [1.28, 1.82]	1.40 [0.99, 1.63]	0.006	1.63 [1.33, 1.76]	1.48 [1.26, 1.65]	0.54
Monocyte count	0.43 [0.30, 0.47]	0.43 [0.31, 0.46]	0.37 [0.24, 0.45]	0.059	0.45 [0.32, 0.51]	0.41 [0.25, 0.47]	0.576
Hemoglobin	112.00 [102.00, 122.00]	112.00 [102.00, 122.00]	112.00 [102.00, 119.00]	0.455	112.00 [103.00, 123.00]	114.00 [112.00, 123.00]	0.311
Neutrophil count	3.98 [2.84, 4.74]	4.06 [2.90, 4.92]	3.20 [2.23, 4.38]	0.025	4.06 [2.82, 4.62]	3.62 [2.97, 4.49]	0.564
NLR	2.67 [1.74, 3.47]	2.37 [1.63, 2.97]	10.00 [9.00, 11.00]	<0.001	2.35 [1.72, 2.82]	11.00 [9.00, 12.00]	<0.001
PLR	156.52 [123.27, 180.89]	146.19 [118.82, 171.15]	280.00 [230.00, 356.00]	<0.001	149.72 [119.02, 168.10]	253.00 [222.22, 314.00]	<0.001
LMR	6.80 [4.66, 9.18]	7.22 [5.30, 9.41]	2.91 [1.64, 3.75]	<0.001	7.59 [5.60, 9.41]	3.72 [2.63, 4.66]	<0.001
PNR	63.11 [49.17, 85.37]	63.11 [46.68, 79.01]	104.06 [76.35, 140.00]	<0.001	63.11 [50.83, 78.84]	97.71 [70.83, 122.36]	<0.001
Fibrinogen	4.28 [3.14, 5.56]	3.95 [3.01, 5.00]	6.72 [6.13, 7.18]	<0.001	4.11 [2.99, 5.25]	6.39 [6.05, 7.04]	<0.001
Albumin	39.40 [36.50, 41.20]	39.40 [36.30, 41.00]	39.30 [36.20, 40.80]	0.822	39.40 [37.23, 41.20]	40.10 [38.30, 42.20]	0.343
GGT	23.00 [15.00, 29.55]	22.00 [15.00, 29.55]	29.55 [19.00, 37.00]	0.012	24.00 [15.00, 29.55]	20.00 [15.00, 29.55]	0.42
Globulin	31.20 [28.50, 33.02]	31.20 [28.48, 33.00]	31.30 [29.40, 34.90]	0.165	31.20 [28.52, 32.27]	31.20 [28.70, 32.30]	0.887
A/G	1.26 [1.15, 1.39]	1.26 [1.15, 1.39]	1.26 [1.11, 1.32]	0.343	1.26 [1.19, 1.41]	1.29 [1.21, 1.39]	0.823
PNI	47.55 [43.50, 49.66]	47.55 [43.50, 49.75]	47.20 [42.00, 48.00]	0.245	47.55 [43.60, 50.44]	48.10 [46.10, 49.85]	0.52
NAR	0.26 [0.17, 0.34]	0.23 [0.15, 0.31]	1.27 [0.98, 1.51]	<0.001	0.25 [0.15, 0.32]	1.12 [0.58, 1.44]	<0.001

Data are shown as median [interquartile range] or n (%).

ADC, adenocarcinoma; AdCa, adenosquamous carcinoma; A/G, albumin-to-globulin ratio; CA125, cancer antigen 125; CA199, cancer antigen 199; CCRT, concurrent chemoradiotherapy; CEA, carcinoembryonic antigen; FBG, fasting blood glucose; FIGO, International Federation of Gynecology and Obstetrics; GGT, γ-glutamyltransferase; IQR, interquartile range; LMR, lymphocyte-to-monocyte ratio; LND, lymph node dissection; NAR, neutrophil-to-albumin ratio; NLR, neutrophil-to-lymphocyte ratio; PLR, platelet-to-lymphocyte ratio; PNI, prognostic nutrition index; PNR, platelet-to-neutrophil ratio; SCC, squamous cell carcinoma.

### Selection of Candidate Variables Using Different ML-Based Algorithms

**Figure 2:**
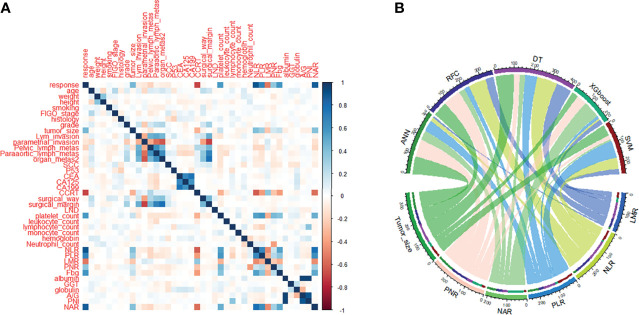
Variable screening and weight allocation. **(A)** Correlation matrix analysis of candidate features. **(B)** Weight distribution of the candidate variables of each ML-based model. ADC, denocarcinoma; A/G, albumin-to-globulin ratio; ANN, artificial neural network; CA125, cancer antigen 125; CA199, cancer antigen 199; CCRT, concurrent chemoradiotherapy; CEA, carcinoembryonic antigen; CIC, clinical impact curve; DCA, decision curve analysis; DT, decision tree; FIGO, International Federation of Gynecology and Obstetrics; GGT, serum γ-glutamyltransferase; LMR, lymphocyte-to-monocyte ratio; LND, lymph node dissection; NAR, neutrophil-to-albumin ratio; NLR, neutrophil-to-lymphocyte ratio; PLR, platelet-to-lymphocyte ratio; PNI, prognostic nutrition index; PNR, platelet-to-neutrophil ratio; RFC, random forest classifier; ROC, receiver operating characteristic curve; SCC, squamous cell carcinoma; SVM, support vector machine; XGboost, eXtreme gradient boosting.

Candidate covariates of each algorithm were filtered and 30 were included in the correlation analysis between outcome and independent variables. rNACT was significantly correlated with inflammatory factors and clinical variables, namely, platelet-to-lymphocyte ratio (PLR), neutrophil-to-lymphocyte ratio (NLR), neutrophil-to-albumin ratio (NAR), lymphocyte-to-monocyte ratio (LMR), and tumor size (**Figure 2A**). PLR, LMR, and NAR were important factors in the ML-based model (**Figure 2B**). Consistent with the results of correlation analysis, the 5 top-ranked predictors were PLR, NLR, NAR, LMR, and tumor size.

### Construction of ML-Based rNACT Predictive Model

**Figure 3:**
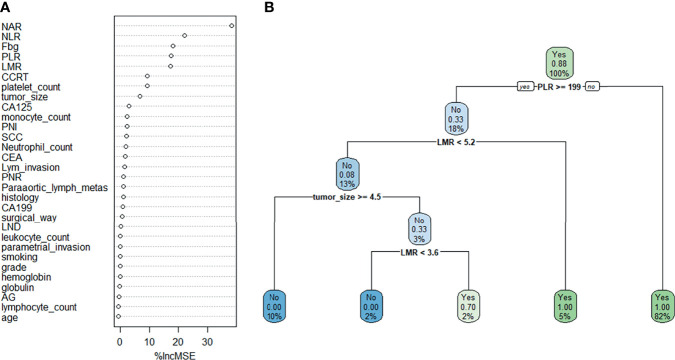
Visualization of the predictive model based on ML-based algorithm. **(A)** RFC model. **(B)** DT model. Candidate factors associated with rNACT were ordered using the RFC algorithm **(A)**, and the prediction node and weight were allocated with the DT algorithm **(B)**.

Random forest classifier (RFC) and decision tree (DT) are commonly used ML-based algorithms in supervised learning. The RFC model was constructed using the formula I(X = x_i_) = −log_2_P(x_i_), where I(X) is the information for candidate variables and P(x_i_) is the probability of x_i_ (**Figure 3A**). Thirty variables were ordered according to the mean decrease in Gini index (**Supplementary Table 1**); the top 10 ranked variables were used to construct the optimal RFC prediction model, which included PLR, NLR, NAR, LMR, and tumor size. Inflammatory factors, PLR, LMR, and tumor size served as irreplaceable weights at DT branches (**Figure 3B**). Using the iterative algorithm of supervised learning, both RFC and DT models were used for rNACT prediction.

### Comparison Among ML-Based Models

**Figure 4:**
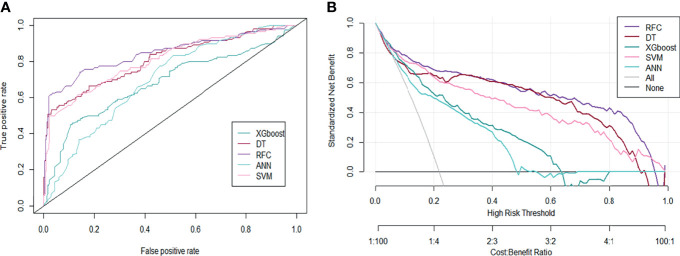
Predictive performance of candidate models based on the ML-based algorithm. **(A)** Area under the ROC curve for 5 ML-based models. **(B)** DCA for the 5 ML-based models. ANN, artificial neural network; DT, decision tree; RFC, random forest classifier; SVM, support vector machine; XGboost, eXtreme gradient boosting.

Based on the iterative analysis of baseline characteristics, we used 5 supervised learning models for NACT risk assessment and to optimize predictive performance. As expected, the RFC model was better able to distinguish between LACC patients in the rNACT and non-rNACT cohorts. The AUCs of the RFC model reached a plateau when *** variables were introduced, indicating that the RFC model had the highest predictive accuracy, followed by DT, artificial neural network (ANN), support vector machine (SVM), and eXtreme gradient boosting (XGBoost) models (**Figure 4A**). The predictive performance of ML-based models is summarized in **Table 2**. Consistent with the results of the ROC analysis, the RFC model also showed a robust predictive performance in the DCA (**Figure 4B**).

**Table 2:** Receiver operating characteristic curve analysis for predicting response to neoadjuvant chemotherapy in each machine learning-based model.

Model	AUC		Number of candidate variables
	Mean	95% CI	
RFC	0.847	0.286–1.408	8
SVM	0.811	0.245–1.377	9
DT	0.807	0.225–1.389	8
ANN	0.682	0.118–1.246	7
XGboost	0.697	0.113–1.281	9

ANN, artificial neutral network; AUC, area under the receiver operating characteristic curve; CI, confidence interval; DT, decision tree; RFC, random forest classifier; SVM, support vector machine; XGboost, eXtreme gradient boosting.

### Internal Validation of the Optimal Predictive Model

**Figure 5:**
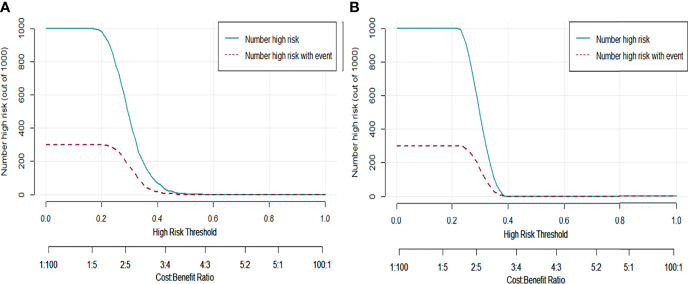
Predictive performance of the RFC model evaluated with the CIC. **(A)** Training set. **(B)** Validation set. The dark green line predicts the probability of poor rNACT, and the purple line shows the number of patients at high risk of non-rNACT.

To further validate the performance of the RFC model, we also used CIC to evaluate predictive accuracy. The CIC analysis revealed rNACT stratification in the training set (**Figure 5A**). This was supported by the risk factors for rNACT identified in the validation set (**Figure 5B**), indicating that the selected features were highly relevant to rNACT.

## Discussion

Inaccurate risk stratification of cancer patients can affect clinical decision-making and outcomes. Given the excellent performance of ML-based algorithms in the classification of rNACT, the RFC, DT, ANN, XGboost, and SVM algorithms were used in our study to establish a predictive model for rNACT in LACC patients. There were 2 major findings to our study. First, we achieved accurate risk stratification of LACC patients who received NACT based on markers of systemic inflammation. Second, we developed and validated a novel ML-based predictive model that is superior to existing prediction algorithms to identify LACC patients who would benefit from NACT.

Systemic inflammation plays a critical role in promoting the progression and metastasis of many cancers (20–23), and was shown to be associated with the development, progression, and metastasis of cervical cancer (24). We therefore examined pre-NACT treatment peripheral blood-related inflammatory marker levels in patients with LACC, and the results were consistent with previous findings (13). As expected, systemic inflammatory markers such as neutrophils, lymphocytes, and platelets and also albumin, C-reactive protein, and other biochemical markers were useful in predicting rNACT in LACC patients. NACT effectively reduces serum levels of tumor markers and NLR and prolongs survival time (25). We compared patients who responded to NACT with those who did not respond and found that in the former, the levels of inflammatory biomarkers were significantly altered compared to before NACT treatment whereas in non-responders, there were no differences between pre- and post-treatment levels. Additionally, we found that PLR, prognostic nutrition index, and LMR were significantly associated with rNACT. Thus, changes in the level of preoperative inflammatory factors can predict the response of LACC patients to NACT.

rNACT was related to NAR and the concentration of fibrinogen, a coagulation factor. The latter has prognostic value in many cancer types, namely, hepatocellular carcinoma (26), gastrointestinal stromal tumors (27), and colorectal cancer (28), and was found to predict the levels of inflammatory factors in our study. In the weighting of the prediction model, it was an index that optimized the accuracy and robustness of rNACT prediction by the RFC model.

Our ML-based model incorporated clinical parameters and laboratory test results according to previous reports (12–14); clinical indicators including larger tumor size and earlier stage were shown to be independent predictors of rNACT (12). We therefore evaluated the predictive accuracy of the models and found that systemic inflammatory markers had a large weight in each model. The RFC model allowed calculation of risk level based on all variables collected from medical records, and yielded the highest predictive accuracy. The RFC uses the bootstrapping resampling technique to reduce variance (29). DCA and CIC analysis were used to assess the predictive performance of the RFC model, and the results showed that the model was able to discriminate between rNACT and non-rNACT cohorts. Thus, the model can be used to identify LACC patients who may benefit from NACT before surgery.

There were some limitations to this study. First, there was selection bias as only patients from a tertiary referral hospital were included. Second, although the predictive model was validated in our study, it may not be applicable to other patient populations; therefore, it must be tested using external data. Third, the ML-based model was only for patients with stage Ib2 to IIa2 LACC; further research is needed to determine whether it can be applied to patients at different stages.

## Conclusion

We developed a ML-based algorithm to identify factors that can predict rNACT in patients with LACC. The model constructed using the RFC had the highest predictive accuracy, with PLR, NLR, NAR, LMR, and tumor size being the most important predictors. Thus, a combination of clinical data and systemic inflammatory markers may aid clinicians in individual risk assessment of rNACT.

## Data Availability Statement

The datasets presented in this study can be found in online repositories. The names of the repository/repositories and accession number(s) can be found in the article/**Supplementary Material**.

## Ethics Statement

The studies involving human participants were reviewed and approved by The Institutional Review Committee of Tongji Hospital, Tongji Medical College, Huazhong University of Science and Technology. Written informed consent for participation was not required for this study in accordance with the national legislation and the institutional requirements. Written informed consent was obtained from the individual(s) for the publication of any potentially identifiable images or data included in this article.

## Author Contributions

YBH and QQZ: study conception and planning, statistical analysis, data interpretation, manuscript drafting, and final approval of manuscript. LRX, XRZ, and YYC: data interpretation and final approval of the manuscript. MFW: study conception and planning, statistical analysis, data interpretation, manuscript drafting, and final approval of the manuscript All authors listed have made a substantial, direct, and intellectual contribution to the work and approved it for publication.

## Funding

This research was supported by the Applied Basic Research Program of WMBST (no. 2019020701011436).

## Conflict of Interest

The authors declare that the research was conducted in the absence of any commercial or financial relationships that could be construed as a potential conflict of interest.

## Publisher’s Note

All claims expressed in this article are solely those of the authors and do not necessarily represent those of their affiliated organizations, or those of the publisher, the editors and the reviewers. Any product that may be evaluated in this article, or claim that may be made by its manufacturer, is not guaranteed or endorsed by the publisher.
