# Supplementary material for: Machine Learning-Assisted Ensemble Analysis for the Prediction of Response to Neoadjuvant Chemotherapy in Locally Advanced Cervical Cancer
Source: Front Oncol. 2022 Mar 29;12:817250. doi: 10.3389/fonc.2022.817250 (PMC9001844; doi:10.3389/fonc.2022.817250)
Supplement: Supplementary file 1 [file Table_1.docx]

sTable1. The candidate variables screening associated with rNACT via RFC algorithm.

| Variables | Mean decrease accuracy | Mean decrease Gini |
| --- | --- | --- |
| Age | -0.590183401 | 0.016887119 |
| Weight | -0.947440664 | 0.010039378 |
| Height | -1.409204845 | 0.032498373 |
| Smoking | -2.14E-35 | 1.87E-16 |
| FIGO_stage | -1.558775952 | 0.002171932 |
| Histology | 1.000500375 | 0.000933333 |
| Grade | -0.045487008 | 0.001383117 |
| Tumor_size | 6.80104457 | 0.655780746 |
| Lym_invasion | 1.414990197 | 0.000126984 |
| Parametrial_invasion | 9.42E-36 | 0.000314478 |
| Pelvic_lymph_metas | -1.342612338 | 0.01930795 |
| Paraortic_lymph_metas | 1.010204466 | 2.30E-16 |
| P53 | -1.218176169 | 0.000484127 |
| CEA | 1.640152202 | 0.044450904 |
| CA125 | 3.03960844 | 0.017791731 |
| CA199 | 0.887988472 | 0.017961088 |
| CCRT | 9.211943393 | 2.968934626 |
| Surgical_way | 0.589585731 | 0.003404762 |
| Surgical_margin | -1.000500375 | 3.25E-17 |
| LND | 0.28628568 | 0.005040736 |
| Platelet_count | 9.203733528 | 2.954915238 |
| Leukocyte_count | 0.075804261 | 0.027111147 |
| Lymphocyte_count | -0.567058477 | 0.031730638 |
| Monocyte_count | 2.373154403 | 0.047377451 |
| Hemoglobin | -0.051735197 | 0.015941418 |
| Neutrophil_count | 1.892915445 | 0.017752582 |
| NLR | 22.08724373 | 18.27402262 |
| PLR | 17.52481251 | 5.168190544 |
| LMR | 17.29017162 | 3.644030396 |
| PNR | 1.168106666 | 0.202026111 |
| Fbg | 18.05302457 | 7.886915934 |
| albumin | -0.673824717 | 0.005831773 |
| GGT | -1.808978917 | 0.010721968 |
| globulin | -0.204659078 | 0.010807636 |
| AG | -0.318403519 | 0.006477602 |
| PNI | 2.33748597 | 0.002217932 |
| NAR | 38.4344245 | 26.05960514 |

Abbreviations: FIGO. The International Federation of Gynecology and Obstetrics. PLR. platelet-to-lymphocyte ratio. NLR. neutrophil-to-lymphocyte ratio. NAR. neutrophil-to-albumin ratio. LMR. lymphocyte-to-monocyte ratio. PNR. platelet-to- neutrophil ratio. PNI. prognostic nutrition index.SCC. Squamous cell carcinoma.ADC. Adenocarcinoma.CEA. Carcinoembryonic antigen.CA125. Glycochain antigen-125.CA199. Glycochain antigen-199.CCRT. concurrent chemoradiotherapy.LND. lymph node dissection.GGT. serum γ- Glutamyltransferase.A/G. albumin-to-globulin ratio.
